# Causes and determinants of inequity in maternal and child health in Vietnam

**DOI:** 10.1186/1471-2458-12-641

**Published:** 2012-08-11

**Authors:** Mats Målqvist, Dinh Thi Phuong Hoa, Sarah Thomsen

**Affiliations:** 1 International Maternal and Child Health (IMCH), Department of Women’s and Children’s Health, Uppsala University, Akademiska sjukhuset, Uppsala, SE-751 85, Sweden; 2 Hanoi School of Public Health, Hanoi, Vietnam; 3 Division of Global Health (IHCAR), Department of Public Health, Karolinska Institutet, Solna, Sweden

**Keywords:** Maternal health, Child health, Equity, Vietnam

## Abstract

**Background:**

Inequities in health are a major challenge for health care planners and policymakers globally. In Vietnam, rapid societal development presents a considerable risk for disadvantaged populations to be left behind. The aim of this review is to map the known causes and determinants of inequity in maternal and child health in Vietnam in order to promote policy action.

**Methods:**

A review was performed through systematic searches of Pubmed and Proquest and manual searches of “grey literature.” A thematic content analysis guided by the conceptual framework suggested by the Commission on Social Determinants of Health was performed.

**Results:**

More than thirty different causes and determinants of inequity in maternal and child health were identified. Some determinants worth highlighting were the influence of informal fees and the many testimonies of discrimination and negative attitudes from health staff towards women in general and ethnic minorities in particular. Research gaps were identified, such as a lack of studies investigating the influence of education on health care utilization, informal costs of care, and how psychosocial factors mediate inequity.

**Conclusions:**

The evidence of corruption and discrimination as mediators of health inequity in Vietnam calls for attention and indicates a need for more structural interventions such as better governance and anti-discriminatory laws. More research is needed in order to fully understand the pathways of inequities in health in Vietnam and suggest areas for intervention for policy action to reach disadvantaged populations.

## Background

Even though maternal and child health is at the core of global public health, projections show that the Millennium Development Goals in this area (MDG 4 & 5) are not going to be met by 2015 [1]. Although there has been substantial progress in improving the survival of mothers and children globally, the changes are not happening fast enough. Additionally there are disadvantaged groups that do not benefit from development to the same extent, resulting in increasing inequity in health [2,3]. It has been shown that as service provision increases and socioeconomic development accelerates health services are actually utilized by those in less need [4]. The better-off women of childbearing age are the ones who access and take advantage of improved services, indicating that inequity is increasing while the overall picture may be that of improvement [5]. Inequity in health is therefore a major challenge to global public health today both for the efforts to reach the Millennium Development Goals and beyond [6].

Vietnam is making progress towards reaching MDG 4 with a drop of under-5 mortality from 58 per 1000 live births in 1990 to 24 per 1000 in 2009 [7]. However, challenges remain, mainly concerning neonatal health and disadvantaged populations [8]. Maternal mortality (MDG 5) has also seen considerable declines from a level of 233 per 100 000 live births in 1990 to 69 per 100 000 live births in 2009 [7]. Contraceptive use and access to antenatal and delivery services have also increased. But just as for the area of child health, the challenges of maternal health lie mainly in closing the widening disparity gaps. Inequities in maternal and child health based on income, asset indices or other measurements, also exist but to a lesser extent [9]. Being a socialist country, the Vietnamese government has long worked to reduce economic disparities in the population. Since the economic reforms known as *doi moi* began in 1986, these efforts have been channeled through different sets of pro-poor initiatives in the health sector. In 2003 the Health Care Fund for the Poor (HCFP; program 139) was launched to provide comprehensive health care to all poor individuals and households [10]. This program provides poor people with a health insurance card to cover costs up to 50 000 VND per year. Despite various successes of these different initiatives it has been noted that economic factors play a relatively minor role as generator of inequity in Vietnam compared to determinants like ethnicity and education in Vietnam [8,11]. People living in remote and mountainous areas, predominantly ethnic minority groups, are being left behind despite efforts from the government to target these groups. Directives like Program 135 (Program for Socioeconomic Development in Communes Faced with Extreme Difficulties) [10], a program that aims to increase living standards in selected communes and includes benefits such as health care free of charge for the communes’ entire populations, have been initiated but have not been fully effective [12] and inequity along ethnic lines in maternal and child survival persist [8,13].

### Inequity and the social determinants of health

**Figure 1:**
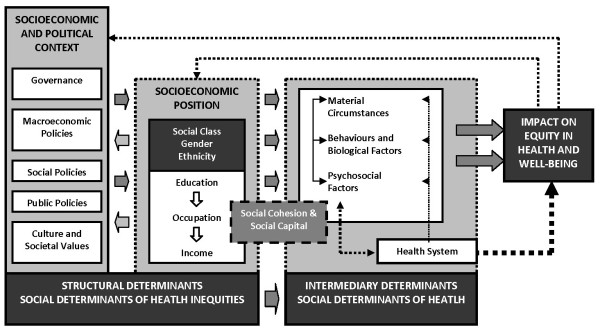
**The Commission on Social Determinants of Health (CSDH) conceptual framework, reproduced with permission **[14].

Inequity, as opposed to inequality, entails a moral dimension when defined as differences in health that are socially produced, systematic in their distribution across the population, and unfair [14]. Health inequality is merely the uneven distribution of health across a population due to natural aging processes, i.e. young people having on average better health than older people [15]. Inequalities become inequitable when such differences are unevenly affected or mediated by social circumstances that are avoidable, such as income or ethnicity, e.g. when access to care is differentiated between people based on social constructs. The Commission on Social Determinants of Health (CSDH) that was set up by the World Health Organization (WHO) has proposed a conceptual framework to orient its work (Figure 1). This framework departs from previous research and aims to aid researchers, policy makers and health planners in their work to reduce health inequity [14].

The key feature of the theoretical framework is the emphasis on social position as the main determinant of inequity. Social position is defined by relations of ownership or control over productive resources and can be captured through social markers such as ethnicity, income, gender and education. The weight and relevance of the assigned social position is influenced by the socioeconomic and political context, including governmental policies, cultural values and the macroeconomic condition of a country. The impact of these structural factors on equity in health and well-being is mediated by behaviours, psychosocial factors, living conditions and access to and quality of care received when encountering the health system [14].

Another important feature of the conceptual framework is its hierarchical approach. In 1984, Mosley and Chen presented a framework for the study of child survival in developing countries [16], emphasizing the need to distinguish between distant and proximate determinants of health. Their combination of socioeconomic factors and biological explanatory variables lay the foundation for a more holistic thinking about the generation or absence of health, which has later been widely adapted [17]. The CSDH framework emphasizes the casual connection between different levels by clearly distinguishing between structural and intermediary determinants [14]. Therefore, we found it highly useful to use this as our theoretical framework in this study of inequity in maternal and child health outcomes in Vietnam.

That inequities in health exist is indisputable, and there are powerful arguments that the disadvantaged populations left behind should be targeted and the disparity gaps closed. Not only is it a moral question of social justice and a matter of human rights [18], but there is also a public health incentive. By reducing disadvantages based on socioeconomic factors that generate ill-health the general health status in a population can be improved, increasing the size of the workforce. Thus, health equity is also a matter of economic development and a prerequisite for long-term economic growth [19]. In order to target the roots of inequity and reduce its scope it is essential to know the causes and determinants of unjust health disparities. Therefore the aim of this review is to map the known causes and contributors to inequity in maternal and child health in Vietnam in order to promote policy action. This process will be guided by the CSDH framework on social determinants of inequity in health. By using this theoretical framework we bring different aspects scattered in the literature together in a structured way and at the same time we assess the applicability of the CSDH framework on maternal and child health in Vietnam. The end result will be a comprehensive look at the issue of inequity in Vietnam in order to engage stakeholders in the country in meaningful dialogue to effectively address the issue.

## Methods

### Search strategy

A systematic, electronic search of academic literature through PubMed and Proquest databases was performed, combining relevant search terms for equity and maternal and child health in Vietnam over the last 10 years (Additional file 1 Annex 1), resulting in a total of 180 peer-reviewed articles. In addition a manual search of “grey literature” was performed through browsing databases of organizations and governmental institutions working in maternal and child health in Vietnam (Additional file 1 Annex 1). Additional articles and publications were identified through citation tracking. All publications and articles were reviewed in full by the first author. Only articles and publications in English were included. Through this search strategy we believe that we have captured most of the relevant publications relating to the aim of the review. Not including publications in other languages than English, especially Vietnamese, is a limitation that must be acknowledged. However, by only including publications in English we ensured transparency and kept the analysis available for an international audience.

### Analysis

A thematic content analysis guided by the CSDH conceptual framework described above was performed on the selected material. In the first step the material was screened and articles and publications describing or containing an analysis of causes, determinants and/or explanation to inequity were kept. This material was then categorized in two steps. First the structural determinant(s) of concern was identified when reading the material and classified into one or more of four categories relating to social position in accordance with the conceptual framework: economic status and occupation, education, ethnicity, and gender. In the next step the intermediary determinant described was identified and classified as belonging to one of four categories, as defined by Solar et al. [14]: material circumstances, behaviours and biological factors, psychosocial factors and the health system. The categories in the framework are not mutually exclusive and therefore there was no conflict of choice when assigning themes. Thirty-one (31) articles and publications were included and presented by this process (Table 1).

**Table 1:** Causes and determinants of inequity in maternal and child health in Vietnam

	**Ethnicity (minorities)**	**Gender (women)**	**Education (low education)**	**Economic status and Occupation (poor)**
**Material Circumstances**	▄ iNutritional status [25-28]	▄ No studies found	▄ Childhood nutrition [53]	▄ Childhood nutrition [54]
	▄ Distance to health facilities [29,38]			▄ Need to take off work [51]
**Behaviors and Biological Factors**	▄ Early childbearing [30]	▄ Women not allowed travelling by themselves [39]	▄ Limited knowledge about contraceptives [31]	▄ Inadequate antibiotics regime due to lack of money [51]
	▄ Cultural taboos towards contraceptives and abortion [30,31]	▄ Women lacking decision making power due to Confucian norms [36,47,48,54]	▄ Less care seeking [51]	▄ No work-cessation before delivery [55]
	▄ Preference for home delivery [24,32-36]	▄ Son preference [45,46]	▄ Less medication use [51]	▄ Time constraints [31]
	▄ Maintenance of traditional health beliefs and rituals [37,38]		▄ Less antenatal care [55]	▄ Self-medication [60]
	▄ Less likely to seek care [13,39]			
	▄ Less likely to treat diarrhea with ORS [40]			
**Psychosocial Factors**	▄ Assimilation policies [41]	▄ Domestic violence [31,49]	▄ No studies found	▄ No studies found
	▄ Discrimination [35,39]	▄ Son preference [48]		
**Health System Factors**	▄ Distance to health facilities [29,38]	▄ Only male staff at CHCs [35]	▄ No studies found	▄ User fees [56,60]
				▄ Indirect costs [39,43,56]
	▄ Low level of cultural competence among health staff [43]	▄ Lack of privacy [50]		▄ No health insurance [62]
	▄ Language barriers [35,42]			
	▄ Discriminatory behaviour [20,31,39]			
	▄ Less covered by health insurance [44]			
	▄ Less contacts within the health system [38]			

## Results

### Ethnicity

Inequity based on ethnicity is well documented in Vietnam. There are 54 different ethnic groups in Vietnam, where of the majority group Kinh constitutes 84% of the population [20]. Ethnic minority groups are disproportionately poor and more often live in remote and mountainous areas [21]. A WHO report from 2005 showed that ethnic minority women have a four times higher risk of maternal mortality compared to Kinh women [22], and a recent study from northern Vietnam indicated ethnic inequity in neonatal survival, independent of household economic status or maternal education [8]. Fertility rates are higher, and the use of modern contraception is lower among minority women as opposed to Kinh women [20,23]. There is also evidence of higher levels of stillbirths [24] and infant mortality among ethnic minority groups [9].

#### Material conditions

Two studies found an association between ethnic minority status and child malnutrition, but neither of the studies had performed appropriate regression or stratification to find possible confounders [25,26]. One of the papers suggested that location is likely to influence the prevalence of stunting [26], and another that living in a rural area more than doubles the risk of a child being stunted [27]. It has also been demonstrated that minority populations consume fewer calories and eat less food rich in starches, lipids and proteins [28].

Ethnic minorities live in remote and mountainous areas to a larger extent than Kinh people. This means that there is a longer distance to health facilities, which in combination with poor infrastructure results in long travel times to reach qualified care. Distance to the closest health facility is associated with neonatal mortality [29]. To tackle this the Vietnamese government has issued directives targeting communes considered to be in extreme need (see above) [10]. Although all inhabitants in the selected communes will benefit from the program, in practice this program will primarily benefit ethnic minority groups.

#### Behaviours and biological factors

Two major contributors to higher fertility rates among ethnic minorities have been suggested; early childbearing and lower rates of abortion due to ideological objections [30]. A qualitative study among H’mong minority women also pointed out cultural taboos as a reason for low rates of modern contraceptive use in this group [31].

The place of delivery is important in relation to health and survival of both mother and child and a number of studies show that ethnic minority mothers are more likely to deliver at home [24], [32-36]. Complex rituals surrounding childbirth and strong traditional beliefs in combination with patriarchal structures have been proposed as the main reason for low facility delivery rates among ethnic minority groups [37,38]. Other factors such as unfamiliarity with the procedures in combination with language barriers and bad attitudes among health staff have also been suggested as explanations for the reluctance to utilize the health system among ethnic minority groups [38,39].

Ethnic minority parents are also less likely to seek care when their children get sick, and they are less likely to report severe illnesses in their children [13]. Mothers belonging to an ethnic minority also use health care less and were less likely to use oral rehydration therapy (ORT) to treat children with diarrhoea [40]. It has however been noted that ethnic minority caregivers possess the ability to accurately recognize danger signs of diarrhoea and that they simultaneously seek care from practitioners of traditional medicine [39] L. The maintenance of traditional healthcare beliefs and practices have been held up as an additional reason for ethnic minorities to be reluctant to use formal healthcare services [38].

#### Psychosocial factors

Belonging to an ethnic minority group can be a source of psychosocial stress due to lower social position. Historically, Vietnamese culture has been favored and there are examples of forceful assimilation policies, promoting Vietnamese culture and Vietnamese language [41]. Even if ethnic minorities today are recognized by the Vietnamese government, and there have been several programs targeting ethnic minority groups in recent years, ethnic status is still an important stratifier in Vietnamese society. Ethnic status is for example specified on identification cards [20] and there are reports of discrimination towards ethnic minorities within the health system [35,39]. For example, there is no information material at local health stations written in any minority language.

#### Health system factors

A dissonant interaction between ethnic minority people and the predominantly Kinh health care workforce, like language barriers and difference in cultural traditions and perceptions has been offered as explanations to discrepancies in quality of care received [35,42]. It has also been pointed out that there is a low level of knowledge and understanding about these culture-specific practices among health staff, exacerbating difficulties in changing practices [43]. Furthermore, there is evidence of discriminatory behaviour of health staff towards ethnic minority groups [20,39], and ethnic minority women say they are mistreated by health staff [35,39]. Ethnic minorities are less well covered by social programs aimed at the poor, like the health care fund for the poor, than their majority counterparts [44], meaning that the minority groups do not fully get the benefits they are entitled to.

### Gender

Patriarchal structures are predominant in most cultures of the world and the Vietnamese culture is no exception, on the contrary there is a strong preference for the hegemonic male. Vietnam is a country influenced by Confucianism, especially in the northern parts of the country where Chinese culture is more predominant. According to the teachings and traditions of Confucius, it is the son of the family who will inherit family resources and preserve the family line in the future. It is also the son who is allowed to make sacrifice to the ancestors and looks after their graves [45]. Thus, a son has the responsibility of looking after the well-being of deceased relatives, and failing to have a son is considered disrespectful to one’s ancestors. This strong preference for sons, for cultural, economic and social reasons, has been one of the driving forces of an increasing sex ratio at birth in recent years [46].

#### Material conditions

No studies found.

#### Behaviors and biological factors

Son preference and the resulting patriarchal structures have implications for equity in health beyond an increasing sex ratio at birth. Gender norms that do not allow a woman to travel on her own [39,47] may result in unnecessary delays and an underuse of health services proportionate to their need.

#### Psychosocial factors

Son preference also affects the psychosocial well-being of women through their implicit lower status in society. No studies were found through the initial search method that investigated the effects of these societal structures. There have however been ethnographic studies describing its impact [48]. Domestic violence also contributes to women’s ill health. A higher incidence of perinatal mental disorders has been reported among women in Vietnam who have experienced intimate partner violence and who were fearful of other family members [49]. Low contraceptive use due to fear of domestic violence has also been reported [31].

#### Health system factors

Cultural norms do not only restrict the health care seeking behaviour of women. Characteristics of the health system itself may also cause gender-related barriers to health. For example, lack of privacy [50] and an insufficiency of female staff [35] have been shown to refrain women from seeking reproductive health care in Vietnam.

### Education

Maternal education has long been considered an important determinant for maternal and child health. Education has an effect on health on many levels, either directly through increased knowledge about danger signs and disease patterns [51] or indirectly through a deeper understanding of health system structures and a higher ability to adapt to change [52]. In addition to individual education level of the mother, it has been argued that the educational level of the whole family and even the general education level of the community has an effect on health [53].

#### Material conditions

Adult educational level has been shown to have an impact on childhood nutrition in Vietnam [53,54].

#### Behaviours and biological factors

Health care seeking and utilization is highly influenced by educational levels in Vietnam [51]. Limited knowledge of danger signs both during pregnancy and in the neonatal period have been shown to contribute to health seeking delays and increase the risk of adverse outcomes for mother and child [52]. However, no studies supporting the notion that mothers with less education should have more limited knowledge about danger signs were found. On the contrary, studies showed no difference in knowledge level concerning disease symptoms between education groups [39,51,55]. The influence of education on health care seeking behaviour must thus have other mechanisms than low understanding of danger signs that still needs to be explored. The use of medication and level of compliance were on the other hand affected by education level [51] and a limited knowledge about contraceptives has also been shown to reduce contraceptives use [31].

#### Psychosocial factors

No studies investigating whether low education causes psychosocial stress in Vietnam were found.

#### Health system factors

No studies indicating that educational level would be of importance in patients’ interaction with the health system was found. However, it is well accepted that higher education leads to a better understanding of how complex organizations function and thus facilitate a better use of them [52], and it is reasonable to assume that this is the case in Vietnam as well.

### Economic status and occupation

Economic status has been the main structural determinant for inequalities in health in the past decades. The poor are vulnerable to ill health due to many co-varying factors, both through living conditions as well as through ability to pay. In Vietnam, a recent report from UNICEF showed considerable differences in maternal and child health due to economic status. In Vietnam, there are now user fees in the health care system, both official as well as unofficial [56]. However, if a family is poor, it can obtain a certificate from the authorities stating their low economic capacity thereby avoid paying the official fees.

Economic status can be measured in different ways. Income has been widely used, but is uncertain in many low-and middle-income countries due to its potential irregularity, with for example seasonal variation and resulting heaping behaviours [57]. Expenditure has instead been proposed as an option, with the argument that a household’s, and especially poor households’, living expenses are more evenly distributed. This approach is of course crippled by the long-term savings for major investments and a strong recall bias, and is thus not robust enough [58]. Asset indices have on the other hand commonly been used as to provide a more lasting and accurate picture of household economic status [58]. The different ways used of measuring economic status sometimes make it difficult to compare studies, but for this literature review they were all considered valid.

In the CSDH framework occupation is also proposed as a structural determinant. Occupational hazards may also be a source of inequity, but no specific studies correlating working conditions to maternal and child health were found. The relationship between occupation and income was however highlighted in some studies included in this category.

#### Material conditions

Living conditions are a direct consequence of economic status and it has been shown that childhood nutritional status may be directly correlated to living conditions [54]. The need for combining work with childcare also affects health-seeking behaviour. Working women report that they cannot afford to take time off work in order to go to the clinic for themselves or their children [51]. This is also related to the fact that women are the primary caregivers for children in Vietnam, as in many parts of the world, even if they work full-time.

#### Behaviours and biological factors

Economic considerations shape much of our behaviours. In relation to maternal and child health much of the literature is centered on care seeking [59], both in terms of actual costs of going to the health station or the hospital, as well as the loss of income limiting health care seeking of poor families and poor mothers who have to work right up until delivery due to economic necessity [55]. Furthermore, there is evidence of time constraints as a reason for not getting appropriate contraceptives [31], and an increased risk of self-medication among the poorer segments of society [60].

#### Psychosocial factors

No studies on the impact of low economic status on psychosocial stress in Vietnam were found.

#### Health system factors

The direct cost of becoming ill is a major problem for an equitable society. In recent years the principal means of financing the healthcare system in Vietnam is through out-of-pocket payments since government spending is not enough to cover all healthcare costs [56,59,61]. There are also a substantial amount of additional costs associated with seeking health care, both through transportation to health facilities and through informal costs to different actors in the system [39,43,56]. The risk of catastrophic spending for the poorest is considerable with this system [59,61], especially since a large proportion of the poor remain uninsured [62].

## Discussion

We have mapped the causes and determinants of inequity in maternal and child health in Vietnam through a systematic review of published literature. By applying the CSDH conceptual framework to the literature we have uncovered factors that generate and sustain inequities in health in a structured way. This approach was feasible and pragmatic and allowed for the identification of research gaps. The thematic content analysis approach has its limitations and it can be argued that we have not captured the full picture, but the searches performed have been extensive and we believe that we have covered the aim satisfactorily.

Some of the determinants found in the review are recognized factors influencing health outcomes; the impact of user fees [56,60], low health care seeking among mothers with low education, and cultural traditions are well-known creators of inequity. More unexpected findings were the influence of informal fees, knowledge about danger signs not being related to education level [39,55] and the many testimonies about discrimination and negative attitudes from health staff towards women in general and ethnic minorities in particular [20,39]. The roles and pathways of these intermediary determinants need to be further explored.

We found few studies that used a theoretical framework for a deeper understanding of what generates inequitable health outcomes. Instead, many studies stratify their outcomes by economy and education, and to some extent also by gender and ethnicity, but few go beyond this to look at why and how this inequity is mediated. By moving beyond proximate determinants of health, like health-seeking behaviour and health systems issues, we are able to look at the root causes of inequities. For example, it is not surprising that those who are poor have difficulty accessing services that cost money. What is interesting is that even when economic barriers to services are theoretically removed (as in Vietnam), some groups still access services less than others. In order to understand the reasons for this we must look at certain key elements in the context such as governance, macroeconomic policies, cultural and gender norms, and social policies and see how they are mediated by factors such as social position (which is measured with proxy variables such as education, income and occupation) [11]. By applying the CSDH structural framework to the published literature we are able to illustrate the different pathways through which structural determinants affect health equity. Doing so allows us to identify what kinds of policy-level changes may be necessary to affect real change in health equity. The CSDH framework also proved to be highly useful by providing a clear structure to the analysis and making the information accessible. It was well suited for the area of maternal and child health.

One example of a macro-level contextual factor affecting health equity in Vietnam is corruption. Informal payments are maybe the most researched aspect of corruption and something that heavily affects the quality of care given [63]. Informal payments make up a large part of health financing in many low- and middle-income countries [64-67], and it has been reported elsewhere that health staff get as much as five to ten times their official salary through unofficial charges [65,66]. The impact of informal fees is most devastating for the poor, since it not only increases the burden of payment but also weakens the effects of exemption policies [68]. As such, it generates barriers to the access of health care and creates inequity in a very direct way. Informal fees and charges also limit governments’ possibilities to act on inequity, not only through by-passing pro-poor initiatives, but by also undermining the ability to generate resources and regulate financing of the health sector [69], which will disfavour the most disadvantaged groups. In Vietnam the system of unofficial fees or “allowances” is wide-spread in society [70] and the health sector is no exception [38,71]. There is however a lack of studies quantifying the extent of informal fees in the Vietnamese health system and its impact on both health staff and patients. In order to tackle inequity this corruption must be addressed.

Discrimination, defined as treatment or consideration of a person based on group belonging rather than on personal merit, is another intermediary determinant of health that needs to be addressed in Vietnam. Discrimination is closely linked to social position, which in turn has been found to be one of the most important structural determinants of health [11]. Social position, or class, is linked to both economic resources and power, which is most evident in the ability to influence the political system. Those who have less power, both politically and economically, have generally a lower social position and worse health. The results of this review indicate that this link is mediated by the behaviours of health care personnel who likely come from another social class and are unable to separate their discriminatory attitudes from their work as professionals. Negative attitudes from health staff may deter women form seeking care [31] and may lead to doctor’s delays [72] and prevent good practice [73]. Negative attitudes towards ethnic minorities among health staff in Vietnam have been reported [39,47], but no intervention studies trying to change such behaviour was found. In fact, there was a general lack of research on discrimination in health care in Vietnam. Women, female sex workers, and adolescents have similarly been shown to suffer from negative effects of power and economic status on behalf of health care provider behaviours in Vietnam and elsewhere [74-77]. One possible solution to this problem is to train and employ more members of ethnic minorities in health care. Another is to raise awareness about discrimination and its effects on health among health care personnel, a strategy that has been shown to be successful in other settings when appropriate pedagogical methods are used [78,79]. As a strong determinant of inequity, discrimination by health care personnel should receive more attention by the government of Vietnam.

Discrimination and corruption are issues closely linked to social capital. In the CSDH theoretical framework social capital/cohesion is placed as an overarching entity involving both structural and intermediary determinants [14]. The concept of social capital has been debated by scholars over the past years, but regardless of the different definitions and applications made there is a common understanding that social capital is trying to capture the influence of social relationships. In an attempt to further organize the concept of social capital Szreter and Woolcock distinguish between bonding, bridging and linking social capital [80]. When it comes to inequity the latter two are of major concern. Discrimination can thus be considered an example of low bridging social capital, which deals with the interactions between individuals that acknowledge that they are not similar in term of social characteristics, whereas corruption can be considered to be a manifestation of low linking social capital, which deals with the interaction between individuals or groups at different levels of institutionalized power. When the deficit of both bridging and linking social capital is at hand, as is the case for ethnic minorities in Vietnam, the effects on inequity are strengthened and may explain the explicitly vulnerable and disadvantaged position of these groups.

## Conclusion

There is still much to be done to ensure equitable health care in relation to maternal and child health in Vietnam. Inequities in health exist based on education, household wealth, place of residence and ethnicity. These inequities are mediated through a complex web of different factors that we have tried to outline in this review and only through a comprehensive approach will policy action be effective. In line with its socialist ideology the Vietnamese authorities have initiated many reforms and programs to target the poor and the ethnic minority groups. These policies have primarily been based on geography or put a lot of responsibility on the individual to take part of existing benefits [56] with varying degrees of success [44,81,82]. There is therefore an increasing need for the authorities in Vietnam to embrace the call from CSDH that reducing inequities in health is primarily the responsibility of governments [14] and that health policies must be extended to cover disadvantaged groups with better efficiency [56].

## Competing interests

The authors declare that they have no competing interests.

## Authors’ contributions

All authors conceived and designed the study. MM and DPH reviewed the material. MM wrote the first draft and DPH and ST made significant contributions to the write-up. All authors have seen and approved the final version of the manuscript.

### What is already known on this subject

▄ There are considerable inequity in maternal and child health in Vietnam.

▄ This inequity has to a large extent been explained by geographical location and difficulties for disadvantaged groups to access health care.

### What this study adds

▄ We provide a method of identifying causes and determinants of inequity by the application of the conceptual framework developed by the Commission of Social Determinants of Health.

▄ Results show a multi-facetted picture of the pathways to inequity and suggest additional areas for intervention compared to what has been traditionally addressed by the Vietnamese government.

▄ Corruption and discrimination are important causes of inequity that have been neglected in research and policy in Vietnam.

## Pre-publication history

The pre-publication history for this paper can be accessed here:

http://www.biomedcentral.com/1471-2458/12/641/prepub

## Supplementary Material

Additional file 1 ** Annex 1.** Search strings used in Pubmed and Proquest. Click here for file
