# Supplementary material for: Causes and determinants of inequity in maternal and child health in Vietnam
Source: BMC Public Health. 2012 Aug 11;12:641. doi: 10.1186/1471-2458-12-641 (PMC3534083; doi:10.1186/1471-2458-12-641)
Supplement: Additional file 1 — Annex 1. Search strings used in Pubmed and Proquest. [file 1471-2458-12-641-S1.doc]

**Annex 1.**

**Search strings used in Pubmed and Proquest**

**1.1. Equity**

**MESH terms:** Healthcare Disparities[MH] OR Health Status Disparities[MH] OR Universal Coverage[MH] OR Vulnerable Populations[MH] OR Poverty[MH] OR Rural Population[MH] OR Residential Mobility[MH] OR Emigration and Immigration[MH] OR Minority Groups[MH] OR Religion[MH] OR Hinduism[MH] OR Islam[MH] OR Christianity[MH] OR Ethnic Groups[MH] OR Adolescent[MH] OR Social Class[MH] OR Socioeconomic Factors[MH] OR Insurance Coverage[MH] OR Health Resources[MH] OR Health Manpower[MH] OR Equipment and Supplies, Cost, Gender[MH]

**Free terms:** equity OR inequity OR inequities OR equality OR inequality OR inequalities OR disparity OR disparities OR “universal coverage” OR “disadvantaged group” OR “disadvantaged groups” OR “disadvantaged population” OR “disadvantaged populations” OR ”vulnerable population” OR “vulnerable populations” OR “sensitive population” OR “sensitive populations” OR poor OR poverty OR indigent OR indigency OR self-employed OR ”informal sector” OR ”informal sectors” OR low-income OR unemployed OR slum OR rural OR urban OR ”agricultural worker” OR ”agricultural workers” OR ”agricultural labor” OR ”agricultural labors” OR farmer OR farmers OR farmworker OR farmworkers OR migration OR migrations OR migrant OR migrants OR emigrant OR emigrants OR immigrant OR immigrants OR minority OR minorities OR ethnicity OR ethnic OR religion OR religious OR tribal OR scheduled OR hindu OR islam OR muslim OR muslims OR Christian OR Christianity OR adolescent OR adolescents OR youth OR youths OR teen OR teens OR teenager OR teenagers OR social class OR social classes OR caste OR castes OR socioeconomic OR “financial constraints” OR “geographical constraints” OR “social norms” OR “health workers attitude” OR “health workers attitudes” OR “health resource” OR “health resources” OR “human resource” OR “human resources” OR infrastructure OR transportation OR supplies OR equipments OR devices OR education OR discriminatory OR discrimination OR uninsured OR insured OR ”insurance status” OR ”insurance coverage” OR "health insurance" OR homeless OR "HIV positive" OR malnutrition OR "drug abuse" OR "drug abstinence" OR "drug addiction" OR cost OR costs OR expense OR expenses OR expenditure OR disability OR handicapped OR disabled OR stigma OR sex ratio OR boys OR girls OR remote OR marginalisation

**1.2 Maternal Health**

**MESH terms:** [Maternal Health Services](http://www.ncbi.nlm.nih.gov/mesh/68008427)[MH] OR Maternal Welfare[MH] OR Maternal Mortality[MH] OR Contraception[MH] OR Family Planning Services[MH] OR Pregnancy[MH] OR Abortion, Induced[MH] OR Abortion, Spontaneous[MH] OR Prenatal Care[MH] OR Delivery, Obstetric[MH] OR Postnatal Care[MH]

**Free terms: “**maternal health care” OR “maternal healthcare” OR “maternal health” OR “maternal services” OR “maternal mortality” OR “maternal mortalities” OR “maternal morbidity” OR contraception OR “birth control” OR “fertilization control” OR “family planning” OR “planned pregnancies” OR pregnancy OR gestation OR abortion OR “pre-natal care” OR “pre-natal visit“ OR pre-natal visits” OR “prenatal care” OR “prenatal visit” OR “prenatal visits” OR “antenatal care” OR “antenatal visits” OR “antenatal visit”OR “obstetric delivery” OR “obstetric deliveries” OR “delivery care” OR “facility-based delivery” OR “skilled birth attendance” OR “postnatal care” OR “post-natal care” OR “postpartum program” OR “postpartum programs” OR “safe motherhood” OR “traditional birth attendant” OR traditional birth attendants”

**1.3. Child health**

**Mesh terms:** pediatrics[MH] OR child mortality[MH] OR infant mortality[MH] OR perinatal mortality[MH] OR neonatal mortality[MH] OR neonate[MH] OR newborn[MH] stillbirth[MH] OR premature birth[MH] OR prematurity[MH] OR Asphyxia Neonatorum[MH] OR low birth weight[MH] OR measles[MH] OR malnutrition[MH] OR Congenital Abnormalities[MH] OR breast feeding[MH] OR immunization[MH] OR vaccination [MH]

**Free terms:** “MDG 4” OR “millennium development goal four” OR “millennium development goal 4” OR pediatrics OR “child health” OR “child mortality” OR “child deaths” OR “child death” OR “under-five mortality” OR “under-five death” OR “infant mortality” OR “infant deaths” OR “infant death” OR “neonatal mortality” OR “neonatal deaths” OR “neonatal death” OR “newborn mortality” OR “newborn deaths” OR “newborn death” OR “child survival” OR “newborn survival” OR “perinatal outcomes” OR “neonatal outcomes” OR stillborn OR premature OR “birth asphyxia” OR “umbilical infection” OR intra-partum OR postpartum OR malformation OR “breast feeding” OR immunization OR vaccination OR IMCI OR PMTCT OR KMC OR “kangaroo mother care” OR skin-to-skin

**Web resources scanned for ”grey literature”**

- <http://www.un.org.vn/en>
- [http://www.gso.gov.vn](http://www.gso.gov.vn/)
- <http://dosei.who.int/>
- [http://www.searo.who.int](http://www.searo.who.int/)
- [http://www.vietnam.unfpa.org](http://www.vietnam.unfpa.org/)
